# Essential Oil of* Ocimum basilicum* L. and (−)-Linalool Blocks the Excitability of Rat Sciatic Nerve

**DOI:** 10.1155/2016/9012605

**Published:** 2016-06-30

**Authors:** Antonio Medeiros Venancio, Francisco Walber Ferreira-da-Silva, Kerly Shamyra da Silva-Alves, Hugo de Carvalho Pimentel, Matheus Macêdo Lima, Michele Fraga de Santana, Péricles Barreto Alves, Givanildo Batista da Silva, José Henrique Leal-Cardoso, Murilo Marchioro

**Affiliations:** ^1^Physiology Department, Federal University of Sergipe, 49.100-000 São Cristóvão, SE, Brazil; ^2^Superior Institute of Biomedical Science, State University of Ceará, Campus of Itaperi, 60.740-000 Fortaleza, CE, Brazil; ^3^Chemistry Department, Federal University of Sergipe, 49.100-000 São Cristóvão, SE, Brazil

## Abstract

The racemate linalool and its levogyrus enantiomer [(−)-LIN] are present in many essential oils and possess several pharmacological activities, such as antinociceptive and anti-inflammatory. In this work, the effects of essential oil obtained from the cultivation of the* Ocimum basilicum* L. (EOOb) derived from Germplasm Bank rich in (−)-LIN content in the excitability of peripheral nervous system were studied. We used rat sciatic nerve to investigate the EOOb and (−)-LIN effects on neuron excitability and the extracellular recording technique was used to register the compound action potential (CAP). EOOb and (−)-LIN blocked the CAP in a concentration-dependent way and these effects were reversible after washout. EOOb blocked positive amplitude of 1st and 2nd CAP components with IC_50_ of 0.38 ± 0.2 and 0.17 ± 0.0 mg/mL, respectively. For (−)-LIN, these values were 0.23 ± 0.0 and 0.13 ± 0.0 mg/mL. Both components reduced the conduction velocity of CAP and the 2nd component seems to be more affected than the 1st component. In conclusion EOOb and (−)-LIN inhibited the excitability of peripheral nervous system in a similar way and potency, revealing that the effects of EOOb on excitability are due to the presence of (−)-LIN in the essential oil.

## 1. Introduction

Aromatic plants of the genus* Ocimum *(Lamiaceae) have been receiving widespread use in folk medicine [1]. This plant has been studied in experimental models of pain [2], inflammation [3], convulsion [4, 5], and other central nervous system disorders [6, 7]. The essential oil (EO) extracted from the leaves of different species of* Ocimum *is rich in several small molecules from different chemical classes (monoterpenes, cyclic sesquiterpenes, and aliphatic secondary alcohols among others) such as d-cadinol, estragole, and linalool (LIN) [8–11]. A new cultivar of the species* Ocimum basilicum* was derived from the Germplasm Bank North Central Regional PI Station (PI 197442, USA) and was called “Maria Bonita” [12]. In this new cultivar linalool content was increased to circa 77% (wild species 40%) and some constituents decreased to undetectable level.

The racemate LIN and its levogyrus enantiomer [(−)-LIN] have shown antinociceptive and anti-inflammatory activities [13, 14] and de Sousa et al. [15], using different models of epilepsy, concluded that LIN enantiomers and racemate have anticonvulsant activity, although with different pharmacological potencies. Previous studies about* Ocimum basilicum* L. (“Maria Bonita”) pharmacological effects showed that (−)-LIN is the principal constituent responsible for the antinociceptive properties of this cultivar [16, 17]. Regarding neuronal excitability, Venâncio et al. [17] showed an inhibition of neuronal excitability in hippocampal slice preparation promoted by the essential oil of* Ocimum basilicum* L. (EOOb) and (−)-LIN and a series of* in vitro* experiments demonstrated direct actions of (−)-LIN on ligand-gated receptors [18–20] and nitric oxide formation [21]. Additionally, Leal-Cardoso et al. [22] showed that racemate LIN concentration-dependently and reversibly blocked the compound action potential (CAP) and the excitability of rat sciatic nerve. However, regarding an essential oil with a rich content of the major constituent, the presence of other constituents besides the major one on the mixture might change (amplifying or partially inhibiting) the effect of the essential oil as related to effect intensity expected solely on basis of the percentage of the major constituent in the oil, as has already been demonstrated [23]. In the case of EOOb, neither the quantitative participation of (−)-LIN on its effect nor the pharmacological potency of (−)-LIN on nerve excitability is known.

Thus, in view of the fact that several EOOb pharmacologic effects might involve alteration of nerve excitability, which makes this effect very relevant, and previous study by Venâncio et al. [17] on hippocampus did not quantitatively evaluate the participation of (−)-LIN on the effect of EOOb (“Maria Bonita”), this work's objectives demonstrate the effects of EOOb on peripheral nerve excitability and the participation of (−)-LIN as its active principle. Additionally, the other studies on peripheral nerves were done with the racemate mixture of linalool and this study also aimed to evaluate the effect of the pure enantiomer (−)-LIN on peripheral nerve excitability.

## 2. Material and Methods

### 2.1. Plant Material and Essential Oil Extraction

Leaves were collected from the cultivation of the* Ocimum basilicum* L. (named “Maria Bonita”) obtained at agricultural research station of Federal University of Sergipe.* Ocimum basilicum* L. was derived from the accession PI 197442 of the Germplasm Bank (North Central Regional PI Station, USA). It is a basil cultivar with a rounded canopy, rose petals, and purple sepals. It is cultivated at Brazilian northeast region [12]. Voucher specimens of the cultivar used in the present study were deposited in the Herbarium of the Federal University of Sergipe (Herbarium ASE) under the number 13162.

The leaves of* Ocimum basilicum* L. were dried in an oven with air renewal and circulation (model MA-037/18) at 40°C until complete dehydration has been achieved. The essential oil was obtained by hydrodistillation in a Clevenger-type apparatus using 100 g of dried leaves. The* Ocimum basilicum* L. leaf essential oil obtained was dried over anhydrous sodium sulphate, producing yields of 4.75 mL (v/w). Gas chromatography-mass spectrometry (GC-MS) and gas chromatography-flame ionization detector (GC-FID) analysis were realized to recognize the compounds of the essential oil of* Ocimum basilicum* L. (EOOb). The EOOb components were separated into aliphatic monoterpenes, cyclic monoterpenes, bicyclic monoterpenes, oxygenated monoterpenes, cyclic sesquiterpenes, bicyclic sesquiterpenes, oxygenated sesquiterpenes, and aliphatic secondary alcohols. The EOOb (Maria Bonita) consisted mainly of linalool (~69.6%), geraniol (~12.6%), 1,8-cineole (~7.5%), neryl acetate (~3.6%), and *α*-trans-bergamotene (~1.2%), representing ~94.5% of total and the list of all compounds of EOOb is found in Venâncio et al. [16].

### 2.2. Animals

In this work we used Wistar rats weighing 250–350 g and the animals were provided by the animal facilities of State University of Ceará. Before the experiments rats were maintained in groups of five per cage and had free access to water and Purina pellets. The experimental protocols here employed were previously approved by the Committee on Ethics on Animal Use of the State University of Ceará (CEUA-UECE, protocol # 06379067-0).

### 2.3. Drugs, Solutions, and Dilutions

Modified Locke's solution was used to provide nutrition of sciatic nerve and its composition (in mmol/L) was NaCl 140, KCl 5.6, MgCl_2_ 1.2, CaCl_2_ 2.2, Tris-hydroxymethyl aminomethane 10, and glucose 10. The pH was adjusted to 7.40 with HCl/NaOH. The (−)-LIN (>98% purity) and dimethyl sulfoxide (DMSO) were purchased from Sigma (USA). For this study, the range doses for EOOb and (−)-LIN were 0.01 to 1.0 mg/mL. The EOOb and (−)-LIN were dissolved in a mixture of DMSO and ethanol of 1 : 10 (v/v) and diluted in Locke's solution in order to obtain the desired doses. The DMSO-ethanol mixture was always added to the control solutions and did not interfere with neuronal excitability [24]. All other salt and drugs were purchased from Sigma (USA) or Reagen (Brazil, PR) and were of analytical grade.

### 2.4. Extracellular Recording of Compound Action Potential

**Figure 1:**
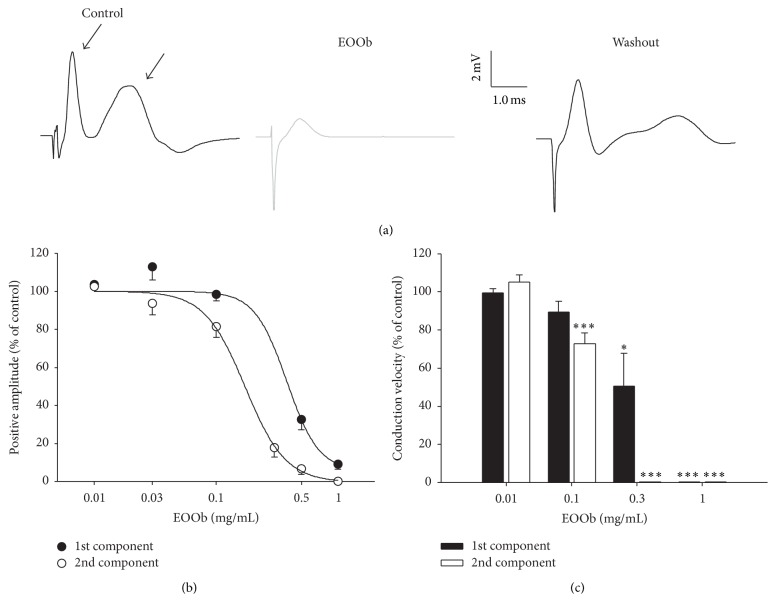
Effects of EOOb on CAP sciatic nerve. Panel (a) shows illustrative traces of CAP waves in control, EOOb, and washout conditions. Panel (b) shows the dose-response curve for 1st and 2nd CAP components and panel (c) shows the conduction velocities of CAP after 60 min EOOb exposure. Data are reported as mean ± SEM. *∗* and *∗∗∗* indicate *p* < 0.05 and *p* < 0.001, respectively (ANOVA followed by Bonferroni's* post hoc* test).

Extracellular recordings of CAP were performed according to Leal-Cardoso et al. [22]. Rat sciatic nerve was mounted in a moist chamber and one of its ends was stimulated with a stimulus isolation unit connected to a stimulator (Model S48, Grass Instruments Co., Quincy, MA, USA). Stimulus and recording platinum electrodes were separated by 50 mm and the evoked CAP was continuously monitored through an oscilloscope (Model 547, Tektronix, Inc., Portland, OR, USA). Computer acquisition hardware was used for data storage and analysis. Between stimulation and recording electrodes, the nerve was immersed in modified Locke's solution used to maintain chamber humidity and to administer the EOOb and (−)-LIN. The nerves were exposed to the substances at least 30 minutes after stabilization of the peak-to-peak CAP amplitude. The period of EOOb and (−)-LIN exposure was set to 60 min and the same interval was used for the washout recovery period. The electrophysiological parameters measured in extracellular recording were the positive amplitude of the 1st and 2nd components of the CAP and the conduction velocity. The amplitudes of the 1st and 2nd components were measured as the maximum positive amplitude in relation to the baseline (see Figure 1). The conduction velocity was estimated according to the equation *v* = *s*/*t*, where *v* is the conduction velocity; *s* is the length of the sciatic nerve (in mm), measured at the end of the experiment starting from the second stimulating electrode; and *t* is the time interval (in ms) between the stimulus artifact and the peak amplitude of each CAP component (first and second components).

### 2.5. Statistical Analysis

Data were expressed as the mean ± SEM. The EOOb and (−)-LIN concentration-response curves on sciatic nerve were fitted by a nonlinear regression sigmoidal curve. To evaluate differences on nerve conduction velocity, we used one-way analysis of variance (ANOVA) followed by appropriate comparison posttest. For all analysis we accepted *p* < 0.05 as statistically significant.

## 3. Results

We investigated the effects of EOOb and (−)-LIN in the CAP of rat sciatic nerve. As seen in Figure 1(a) left panel, the CAP signal shows two waves, named here as 1st and 2nd CAP components. The control values of positive amplitudes and conduction velocities of 1st CAP component were 4.1 ± 0.3 mV and 84.0 ± 2.6 m/s. For the 2nd component the values were 3.1 ± 0.3 mV and 33.5 ± 1.9 m/s (*n* = 48), respectively.

Figure 1 shows illustrative traces of CAP in control, EOOb exposure, and washout conditions. As seen in Figure 1(a), EOOb (0.5 mg/mL) was effective in blocking the 1st and 2nd CAP components of sciatic nerve and the blockade was reversible after washout. After 60 min of exposure, EOOb decreased significantly and in a concentration-dependent manner the CAP amplitudes (Figure 1(b)) and calculated IC_50_ for 1st and 2nd CAP components were 0.38 ± 0.2 and 0.17 ± 0.0 mg/mL, respectively. EOOb also altered the CAP conduction velocity (Figure 1(c)). The 2nd component was significantly reduced in doses equal to or above 0.10 mg/mL and the 1st component at doses above 0.30 mg/mL (*p* < 0.05, ANOVA followed by Dunn's comparison test). Due to the great reduction in CAP amplitude promoted by EOOb 1.0 mg/mL for 1st and 2nd components and 0.3 mg/mL for 2nd component, the conduction velocity of components could not be measured.

**Figure 2:**
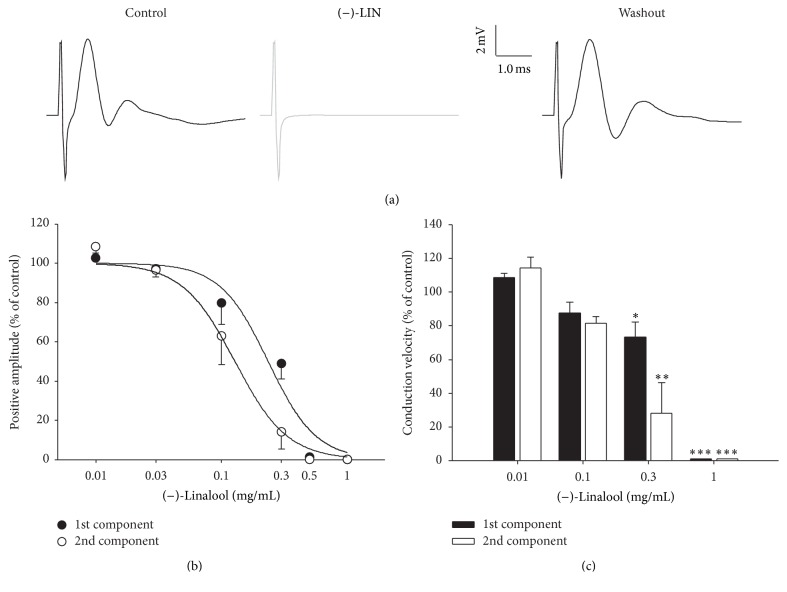
Effects of (−)-LIN on CAP sciatic nerve. Panel (a) shows illustrative traces of CAP waves in control, (−)-LIN, and washout conditions. Panel (b) shows the dose-response curve for 1st and 2nd CAP components and panel (c) shows the conduction velocities of CAP after 60 min (−)-LIN exposure. Data are reported as mean ± SEM. *∗*, *∗∗*, and *∗∗∗* indicate *p* < 0.05, *p* < 0.01, and *p* < 0.001, respectively (ANOVA followed by Bonferroni's* post hoc* test).

Since the main constituent of EOOb is (−)-LIN, we decided to investigate its effects on the conductibility of CAP in sciatic nerve.  Figure 2 shows the CAP in control and (−)-LIN exposure and after 60 min washout. (−)-LIN (0.5 mg/mL, Figure 2(a)), accordingly, reversibly blocked both components of the CAP sciatic nerve in a concentration-dependent manner (Figure 2(b)) with IC_50_ values for the 1st and 2nd components of 0.23 ± 0.1 and 0.13 ± 0.0 mg/mL, respectively. For the CAP conduction velocities, significant inhibition promoted by (−)-LIN was seen from the concentration of 0.3 mg/mL, as shown in Figure 2(c) (*p* < 0.05, ANOVA followed by Dunn's comparison test). Like EOOb, the conduction velocity of 2nd CAP component was more affected than 1st CAP component. Finally, at the concentration of 1.0 mg/mL there was such a reduction in CAP amplitude that the conduction velocities of both components could not be measured.

## 4. Discussion

In this study we described the effects of EO extracted from the leaves of the aromatic plant* Ocimum basilicum* L. and of monoterpenoid (−)-LIN, its major constituent, on the excitability of peripheral nervous system. The EOOb and (−)-LIN showed very similar results in the electrophysiological data described in this work, both being more potent on the blockade of the 2nd component of CAP than of the first. (−)-LIN showed a clear tendency to be pharmacologically more potent than EOOb at a given type of CAP component. This is coherent with the suggestion that (−)-LIN is mainly responsible for the pharmacological effects of EOOb described below, since, in a given concentration of this essential oil, (−)-LIN is diluted by the presence of the other components.

Both EOOb and (−)-LIN showed concentration-dependent effects on CAP amplitude and recovery of its effects after washout. The recovery of its effects is common to some essential oils such as* Croton zehntneri* Pax et Hoffm. and* Lippia alba* (Mill.) N. E. Brown [25, 26] but not for* Croton nepetaefolius* Bail. [23]. Regarding CAP amplitude, the effects of essential oils present different pharmacological potency. At the end of 180 min exposure the threshold dose (dose that produces a significant reduction of CAP peak-to-peak amplitude) of EOCn was 500 *μ*g/mL [23]. For* Lippia alba *(Mill.) N. E. Brown essential oil (EOLa), the threshold dose was 30 *μ*g/mL and complete CAP blockade was achieved in 300 *μ*g/mL of EOLa [26]. For* Croton zehntneri* Pax et Hoffm. essential oil, the reduction was seen at a dose of 100 *μ*g/mL and IC_50_ of 320 *μ*g/mL [25]. The* Alpinia zerumbet* (Pers.) Burtt. et Smith essential oil reduced significantly the CAP amplitude at 300 *μ*g/mL [27]. Different from these studies, the data here presented show the effect of EOOb in both components of CAP. The IC_50_ for 1st and 2nd CAP components (380 and 170 *μ*g/mL, resp.) were similar to other essential oils, excluding* Lippia alba* (Mill.) N. E. Brown essential oil, although the exposure period was smaller (60 min of exposure). These facts indicate faster establishment of EOOb effects in sciatic nerve excitability and conductibility.

Regarding (−)-LIN, the effects on CAP amplitude were similar or lower than other constituents. For estragole and anethole, the IC_50_ for CAP amplitude were ~593 and 220 *μ*g/mL (4.0 and 1.5 mmol/L, resp.) [27, 28]. Leal-Cardoso et al. [22] also showed that racemic mixture of linalool reduced 1st and 2nd CAP components with an IC_50_ of, approximately, 120 and 100 *μ*g/mL (0.75 and 0.64 mmol/L, resp.) and it was similar to the doses found in this work. It is to note in those works that the necessary exposure time (to reach steady state effect) of sciatic nerve to linalool was 180 min and the necessary exposure time in this work was set to 60 min. Thus, it seems that (−)-LIN establishes its effect more rapidly than estragole, anethole, and even the linalool racemic mixture.

Regarding conduction velocity, both EOOb and (−)-LIN were effective in reducing this parameter. The 2nd component seems to be more affected by EOOb than the 1st one, since EOOb reduced significantly the 2nd CAP conduction velocity at 100 *μ*g/mL and the same effect was seen in* Croton zehntneri* Pax et Hoffm. [28]. The (−)-LIN acts in a similar way. The reduction in both conduction velocities was seen at 300 *μ*g/mL (−)-LIN and this fact was seen for other essential oil constituents, such as citral [26] and carvacrol [29]. As seen in the illustrative traces, our CAP is composed of two waves, named here as 1st and 2nd components. The 1st component reflects the electrical activity of the fibers with the largest diameter, predominantly motor. The second component reflects the electrical activity of the fibers with intermediate diameter, predominantly sensory. The greater pharmacological potency on the 2nd CAP component thus suggests this latter type of fiber is more sensitive to EOOb and (−)-LIN than the fibers related to the first component and this effect is observed for many classical local anesthetics.

Although this work did not investigate the mechanism of action of EOOb and (−)-LIN on nerve excitability, we formulate some hypothesis. As shown for several essential oils and constituents, they could act on excitability by the blockade of ion channels responsible for action potential generation, for example, sodium channels. Estragole is a majority constituent of* Croton zehntneri* Pax et Hoffm. essential oil and it was shown to inhibit Na^+^ current of dorsal root ganglia (DRG) in a concentration-dependent way [28]. Joca et al. [29] showed that carvacrol, present in essential oils of genera* Origanum* and* Thymus*, blocked the generation of action potential in intact DRG and reduced the Na^+^ current in dissociated DRG neurons. In a different way, 1,8-cineole, present in EOCn, blocked the generation of action potential with a depolarization of resting potential of intact superior cervical ganglion and alteration of kinetic parameters sodium channel inactivation [30, 31]. Also, Leal-Cardoso and coauthors [22] showed that racemic linalool blocked the generation of action potential (AP) and reduced the amplitude of Na^+^ current in DRG neurons. Additionally, the effects of linalool on the nervous system were studied by means of* in vitro *[18, 20, 21] and* in vivo *[14–17] preparations. The* in vitro* studies of Elisabetsky's group demonstrated a preferential action of linalool on glutamatergic related targets. The monoterpenoid inhibited glutamate uptake and release in cortical synaptosomes [18] and inhibited MK-801 binding in the rat cortical membranes [19]. These evidences were used to explain the anticonvulsant properties of linalool revealed in* in vivo* seizure models [32, 33]. Moreover, linalool was shown to interact with the muscle nicotinic acetylcholine receptor [20] suggesting possible interactions with membrane proteins. Thus, it is reasonable to hypothesize that (−)-LIN could act on protein membranes responsible for AP generation, such as Na^+^ channels, as does its racemate or a racemic mixture [22]. However, further experiments are needed to ensure that (−)-LIN could act on Na^+^ channels or other voltage-gated ion channels related to excitability process.

## 5. Conclusions

In this study we described the effects of the essential oil extracted from the leaves of a cultivar of the aromatic plant* Ocimum basilicum* L. developed to have a richer content of a pure enantiomer of the monoterpenoid, the (−)-LIN, its major constituent, on the excitability of peripheral nervous system. The EOOb and (−)-LIN inhibited the excitability of peripheral nervous system in a similar way and potency, but revealing a stronger pharmacological potency on the second CAP component which reflects predominant activity of sensory fibers. Additionally we have demonstrated that the effects of EOOb on excitability are due to the presence of (−)-LIN on the essential oil. Since* Ocimum basilicum *L. is greatly used in folk medicine and linalool has several pharmacological activities which may include antiexcitability in its mechanism of action, we believe to have contributed with this study to further investigations on the effects of the* Ocimum basilicum *L., of its essential oil, and of (−)-LIN.
